# Combining Plasma miRNAs and Computed Tomography Features to Differentiate the Nature of Pulmonary Nodules

**DOI:** 10.3389/fonc.2019.00975

**Published:** 2019-10-01

**Authors:** Kexing Xi, Weidong Wang, Yingsheng Wen, Yongqiang Chen, Xuewen Zhang, Yaobo Wu, Rusi Zhang, Gongming Wang, Zirui Huang, Lanjun Zhang

**Affiliations:** ^1^Department of Thoracic Surgery, Sun Yat-sen University Cancer Center, Guangzhou, China; ^2^State Key Laboratory of Oncology in South China, Collaborative Innovation Center for Cancer Medicine, Guangzhou, China; ^3^Department of Medical Oncology, Sun Yat-sen University Cancer Center, Guangzhou, China; ^4^State Key Laboratory of Organ Failure Research, Guangdong Provincial Key Laboratory of Viral Hepatitis Research, Department of Infectious Diseases, Nanfang Hospital, Southern Medical University, Guangzhou, China

**Keywords:** microRNA, pulmonary nodules, prediction model, diagnosis, NSCLC

## Abstract

**Objective:** The purpose of this study was to evaluate the diagnostic efficiency of combining plasma microRNAs (miRNAs) and computed tomography (CT) features in the diagnosis of pulmonary nodules.

**Methods:** Ninety-two pulmonary nodule patients who had undergone surgery were enrolled in our study from July 2016 to March 2018 at the Sun Yat-sen University Cancer Center. A prediction model was established by combining 3 miRNAs (miRNA-146a, -200b, and -7) and CT features to identify the pulmonary nodules of these patients. We evaluated the diagnostic performance of this prediction model for pulmonary nodules using the Receiver Operating Characteristic (ROC) curve.

**Results:** The expression levels of miRNA-146a, -200b, and -7 in early-stage non-small cell lung cancer (NSCLC) patients are significantly higher than those in benign nodule patients. We used these three miRNAs and CT features (pleural indentation and speculation) to establish a prediction model for early-stage NSCLC, with a sensitivity and specificity of 92.9%, 83.3% in the training set, respectively. For the validation process, with the sensitivity of 71.8% and the specificity of 69.2%. For ROC curve analyses, area under the curve (AUC) for tumor identification in the training stage and validation stage were 0.929 and 0.781, respectively.

**Conclusion:** Plasma miRNA-146a, miRNA-200b, and miRNA-7 may be potential biomarkers for the early diagnosis of lung cancer. Our prediction model can help to identify the nature of pulmonary nodules with a relatively high diagnostic efficiency.

## Introduction

Lung cancer is the most common cancer in the world with high morbidity and mortality based on the global cancer statistics from 2012 (1). In all, 80~85% of lung cancer cases are non-small cell lung cancer (NSCLC) (2). To date, the 5-year overall survival of lung cancer is only approximately 15%, despite the advances of new diagnostic techniques and treatments over the last few decades (3). One of main reasons for the poor prognosis of lung cancer is that more than 60% of patients have advanced NSCLC at the time of diagnosis (4). The early diagnosis of lung cancer is vital to improve the overall survival rate and prognosis of lung cancer.

At present, the diagnosis of lung cancer clinically depends on a pathology examination. However, this method requires obtaining tissue from patients, which involves invasive procedures, such as percutaneous needle lung biopsy, bronchoscopy, and video-assisted thoracoscopic surgery (VATS). And a non-invasive test is more valuable, which can reduce the economic burden and surgical wound. Thanks to the increasing widespread use of computed tomographic (CT) scans, some early-stage lung cancer cases can be discovered. Moreover, based on the National Lung Screening Trial (NSLT), it was found that high-risk individuals who received low-dose computed tomographic (LDCT) screening had a 20% reduction in the mortality of lung cancer (5). However, some studies reported that screening CT had a high false positive rate which may lead to unnecessary treatment (5, 6). In the meantime, some tumor markers including Neuron-specific enolase (NSE), Carcinoembryonic antigen (CEA), Squamous cell carcinoma antigen (SCCA), and Cytokeratin-19 fragments (Cyfra21-1), have been widely used in the clinic. However, the specificity and sensitivity of these tumor markers for differentiating malignant or benign pulmonary nodules are limited (7). Furthermore, most of them were increased in advanced-stage lung cancer patients. Thus, it is urgent to find new methods and molecular biomarkers for detecting and diagnosing lung cancer.

MicroRNAs are endogenous noncoding RNAs of approximately−22nt in length (8). They participate in post-transcriptional gene regulation, and may act as potential molecular biomarkers (9). Numerous studies have reported the diagnostic value of miRNAs in pulmonary nodules and that they can serve as novel biomarkers for the early detection of lung cancer. According to the published literature and our previous study, we selected 10 miRNAs (miR-17, -146a, -200b, -182, -221, -205, -7, -21, -145, and miR-210) for our study (10–14). We aimed to measure the differences in expression of miRNAs between benign and malignant pulmonary nodules and then select the target miRNAs in this study. Finally, we combine miRNAs and CT features to diagnosis the nature of pulmonary nodules.

## Materials and Methods

### Patients

In this retrospective study, all 92 patients were enrolled from July 2016 to March 2018 at the Sun Yat-sen University Cancer Center. Patients were collected based on the following eligibility criteria: (1) Patients were diagnosed with a pulmonary nodule by CT before the surgery; (2) Patients diagnosed as stage 0~IIa non-small cell lung cancer (NSCLC) or benign lung disease pathologically after surgery; (3) Patients diagnosed with NSCLC can obtain accurate pathologic staging; and (4) Patients had complete clinicopathologic information. The exclusion criteria in this study were as follows: (1) Patients received radiotherapy or chemotherapy before the surgery; (2) Patients had a second primary tumor; (3) Patients were not pathologically confirmed after surgery; and (4) Patients with stage IIb~ IV lung cancer. The patients selected for the training set and validation sets were according to the time of diagnosis. Patients were staged before the surgery according to the examination including brain MRI, thoracoabdominal CT, even positron emission tomography (PET). Patients were staged after the surgery according to the result of pathology. The pathologic staging of tumor was according to the International Association for the Study of Lung Cancer (8th version). This study was approved by the Ethics Committee of Sun Yat-sen University Cancer Center (NO. B2017-050) and written informed consent was obtained from all patients.

### RNA Extraction and RT-PCR

Five milliliter of venous blood was collected from every patient before the surgery, centrifuged within 2 h at 3,200 rpm for 10 min at 4°C and then stored at −80°C until use.

Total RNA was extracted from 200 μl of plasma samples using miRNeasy Serum/Plasma Kit (Qiagen, USA) according to the manufacturer's instructions, and eluted to a final volume of 14 μl. The total reaction volume for Poly(A) tailing was 25 μl including eluted RNA, 10 μl; cel-miR-39, 1 × 10^9^ copy; 5 × PAP buffer solution, 4 ul; PolyA polymerase (Life, 74225Y/Z), 2~5U; and appropriate RNase-free water to the volume of 25 μl. The reaction conditions for Poly(A) tailing were 37°C for 10~20 min, 65°C for 10 min. Adding a total of PolyA-tailed RNA, 10 μl; RT buffer solution, 2 μl; dNTPs, 2 μl; reverse primers, 20 μM; Omniscript (Qiagen Cat No.205111), 4U and appropriate RNase-free water to the Reverse transcription (RT) reactions, and the final volume is 20 μl. The conditions for reverse transcription (RT) reactions were incubated at 37°C for 1 h, 85°C for 5 min, and terminated at 4°C. The total volume for real-time PCR reaction was 20 μl and contained 1 μl cDNA, 10 μl 2 × SYBR Green Mix, 10 μM forward primers, 10 μM reverse primers and appropriate H_2_O. The conditions for real-time PCR reaction were as follow: 95°C for 3 min; 40 cycles of 95°C for 15 s and 60°C for 35 s; 95°C for 15 s and 60°C for 1 min.

We used the Omniscript RT Kit (Qiagen, Germany) to accomplish reverse transcription (RT) reactions. The SYBR Green Mix (Qiagen, Cat No.208054) was used for real-time quantitative polymerase chain reaction (RT-PCR) analysis to detect the expression levels of miRNAs. The relative expression of miRNAs was analyzed with the 2^−ΔΔ*Ct*^ method (15). The cel-miR-39 was chosen as the inner control.

### Statistical Analysis

Statistical analyses were performed using SPSS software version 21 (SPSS Inc., Chicago, IL). We used the Shapiro-Wilk test for the test for normal distribution. The Wilcoxon rank-sum test was used when the data were not normally distributed. When the data were normally distributed, we used the *t*-test for univariate analysis. The Mann-Whitney *U*-test and *t*-test were used to compare the difference of the expression of miRNAs between benign and malignant pulmonary nodule patients. The *t*-test, Mann-Whitney *U*-test, and Kruskal-Wallis test were used to evaluate the relationship between the expression levels of the plasma miRNAs and clinicopathologic characteristics. The risk score model was built using logistic regression. The Receiver Operating Characteristic (ROC) curve was used to evaluate the diagnostic performance of plasma miRNAs and the risk score model for distinguishing benign and malignant pulmonary nodules. Because of the magnitude of the miRNA levels measured, the results were log transformed for analysis when needed. *P*-values <0.05 was considered to indicate a statistically significant difference.

## Results

### Patient Description

In all, 25 benign pulmonary nodule patients and 67 malignant pulmonary nodule patients were enrolled in this study (Table 1). The age of all participants ranged from 32 to 81 years. The median ages of benign pulmonary nodule patients and NSCLC patients were 57 and 61 years, respectively. In the training set, there were 12 participants in the benign group and 28 patients in the NSCLC group. In the validation set, 13 benign pulmonary nodule patients and 39 NSCLC patients were included. Among the NSCLC patients, including 1 patient of stage 0, 48 patients of stage Ia, 14 patients of stage Ib, and 4 patients of stage IIa. The clinical and imaging features of the patients are shown in Table 1.

**Table 1:** Clinical features of benign pulmonary nodule patients and NSCLC patients (n, %).

	**Training set**		**Validation set**	
**Characteristic**	**Benign (*n =* 12)**	**NSCLC (*n =* 28)**	**Benign (*n =* 13)**	**NSCLC (*n =* 39)**
**Gender**				
Men	4 (33.33)	15 (53.57)	9 (69.23)	17 (43.59)
Women	8 (66.67)	13 (46.43)	4 (30.77)	22 (56.41)
**Age**				
≤ 60	10 (83.33)	14 (50.00)	9 (69.23)	19 (48.72)
>60	2 (16.67)	14 (50.00)	4 (30.77)	20 (51.28)
**Smoking index**				
<400	10 (83.33)	20 (71.43)	8 (61.54)	28 (71.79)
≥400	2 (16.67)	8 (28.57)	5 (38.46)	11 (28.21)
**pTNM stage**				
0~Ia		18 (64.29)		31 (79.49)
Ib		7 (25.00)		7 (17.95)
IIa		3 (10.71)		1 (2.56)
**Differentiation grade**				
Poor		7 (25.00)		6 (15.38)
Moderate		14 (50.00)		25 (64.10)
Well		7 (25.00)		8 (20.51)
Nodule diameter (cm)	1.90 ± 1.25^a^	2.51 ± 1.31^a^	1.40 ± 0.53^a^	1.83 ± 0.83^a^
speculation	0 (0.00)	14 (50.00)	4 (30.77)	21 (53.85)
Pleural indentation	1 (8.33)	13 (46.43)	1 (7.69)	16 (41.03)
Air bronchogram	0 (0.00)	3 (10.71)	2 (16.67)	5 (12.82)
Vessels sign	1 (8.33)	3 (10.71)	0 (0.00)	2 (5.13)

*NSLCL, non-small cell lung cancer; TNM, tumor-node-metastasis*.

a *Data are presented as mean ± SD*.

### miRNA Selection

We detected the levels of 10 miRNAs (miR-17, -146a, -200b, -182, -221, -205, -7, -21, -145, and miR-210) in 28 NSCLC patients and 12 benign subjects in the training stage. The results revealed that the levels of miR-146a, miR-200b, and miR-7 in NSCLC group were statistically higher than those in benign subjects. The *P*-values were 0.021, 0.048, 0.040, respectively. However, for the expression levels of the other seven miRNAs, there were no significant differences between these two groups. The details are shown in Table 2.

**Table 2:** Expression levels of 10 miRNAs between 28 NSCLC patients and 12 benign pulmonary nodule patients [mean ± SD and median (Q25–Q75)].

**miRNAs**	**NSCLC group**	**Benign group**	***P*-value**
miR-17	8.716 (8.054–9.281)	8.117 (7.341–8.390)	0.059^a^
miR-146a	8.371 (7.562–9.085)	7.628 (6.720–7.961)	0.021^a^
miR-200b	6.678 (5.482–7.585)	5.319 (3.916–5.817)	0.048^a^
miR-182	6.898 ± 1.377	6.212 ± 1.187	0.141^b^
miR-221	8.409 ± 1.060	7.800 ± 0.904	0.091^b^
miR-205	6.743 (6.235-8.008)	6.278 (4.866-6.891)	0.157^a^
miR-7	7.312 (6.745-8.346)	6.379 (5.635-6.731)	0.040^a^
miR-21	9.045 (8.165-9.705)	8.347 (7.732-8.909)	0.092^a^
miR-145	8.004 ± 1.000	7.471 ± 0.962	0.127^b^
miR-210	7.756 (6.536-8.569)	6.269 (5.558−7.267)	0.082^a^

*NSCLC, non-small cell lung cancer*.

a *P-value of Mann-Whitney U*,

b *P-value of t-test*.

### Establish a Prediction Model

In the first stage, we selected 3 miRNAs (miR-146a, miR-200b, and miR-7) from 10 miRNAs (miR-17, -146a, -200b, -182, -221, -205, -7, -21, -145, and miR-210) in 28 NSCLC patients and 12 benign subjects. Then, we used logistic regression analyses on variables, including miR-146a, miR-200b, miR-7, gender, age, smoking index, nodule diameter, speculation, pleural indentation, air bronchogram, and vessel sign, to establish a prediction model. The CT features were evaluated by two radiologists independently. A third radiologist made the final decision when the two radiologists disagreed. The following variables were enrolled into the prediction model, including miR-146a, miR-200b, miR-7, speculation, and pleural indentation. The prediction model was described by the following equations: Y = 1.011×miR-146a+0.907×miR-200b-0.795×miR-7+23.109×speculation+4.291×pleural indentation-8.648.

Note: Smoking index is a parameter which is used to quantify cumulative smoking exposure. Usually, multiply the number of cigarettes smoked every day by the years smoked to get the Smoking index.

### Diagnostic Value of the Prediction Model

**Figure 1:**
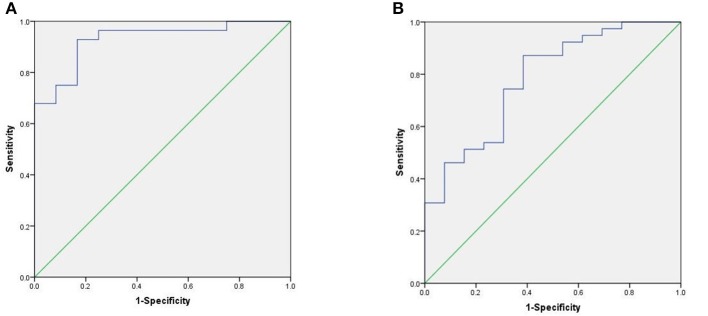
ROC curve of the prediction model. **(A)** ROC curve of training stage (AUC = 0.929). **(B)** ROC curve of validation stage (AUC = 0.781).

**Figure 2:**
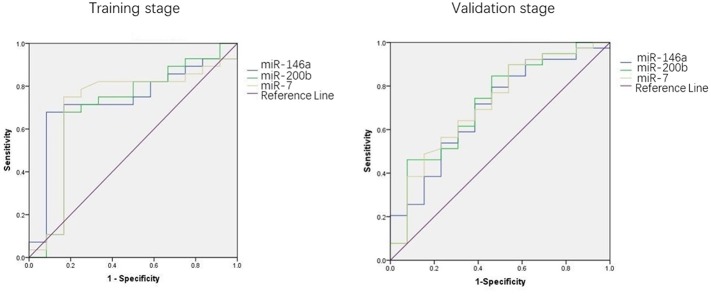
ROC curves of three miRNAs.

We used the ROC curve to assess the efficacy of miRNAs and the prediction model for the diagnosis of pulmonary nodules. In the training set, the AUC, sensitivity, and specificity were 0.732, 67.9, and 91.7% for miR-146a; 0.699, 67.9, and 83.3% for miR-200b; 0.707, 75.0, 83.3% for miR-7, respectively. The AUC of the prediction model was 0.929, with a sensitivity of 92.9%, and a specificity of 83.3% using the optimal cutoff value of 0.4404 (Table 3). At this point, the sensitivity + specificity were considered to be maximal. In the validation stage, 13 benign patients and 39 NSCLC patients were included. The same cutoff value was used to determine the risk score for the cases. The ROC curve analysis of the prediction model allowed us to distinguish early-stage NSCLC from benign pulmonary nodule patients with 71.8% sensitivity and 69.2% specificity (AUC = 0.781), other results were shown in Table 4. The ROC curves of three miRNAs (miR-146a, miR-200b, and miR-7) and the prediction model were shown in Figures 1, 2, respectively.

**Table 3:** The results of miRNAs and the prediction model in the diagnosis of pulmonary nodules in the training set.

**Category**	**AUC**	**Sensitivity**	**Specificity**	**Cutoff value**	**95%CI**	***P***
miR-146a	0.732	67.9%	91.7%	8.0528	0.561~0.903	0.021
miR-200b	0.699	67.9%	83.3%	6.0335	0.503~0.896	0.048
miR-7	0.707	75.0%	83.3%	6.7858	0.511~0.903	0.040
Prediction model	0.929	92.9%	83.3%	0.4404	0.847~1.000	<0.001

*AUC, area under the curve; CI, confidence interval*.

**Table 4:** The results of miRNAs and the prediction model in the diagnosis of pulmonary nodules in the validation set.

**Category**	**AUC**	**95%CI**	***P***
miR-146a	0.696	0.530~0.863	0.035
miR-200b	0.724	0.558~0.889	0.016
miR-7	0.717	0.551~0.883	0.020
Prediction model	0.781	0.636~0.926	0.003

*AUC, area under the curve; CI, confidence interval*.

### Association of the Expression of miR-146a, miR-200b, miR-7 With Clinical Characteristics

We explored the relationship between the expression of these three miRNAs and the patient clinical status in all NSCLC samples. As shown in Table 5, there are no significant connections between the expression of miR-146a, miR-200b, miR-7, and gender, age, smoking index, pTNM stage, or differentiation grade.

**Table 5:** Relationship between clinicopathologic characteristics and miRNAs expressions in NSCLC.

**Characteristics**	**Number**	**miR-146a**	***P***	**miR-200b**	***P***	**miR-7**	***P***
**Gender**			0.620		0.425		0.990
Men	32	8.068 (7.122–8.545)^a^		6.249 (4.145–6.912)^a^		7.312 (5.603–7.525)^a^	
Women	35	8.137 (7.636–8.547)^a^		5.842 (4.592–6.465)^a^		7.230 (6.615–7.565)^a^	
**Age**			0.778		0.792		0.744
≤ 60	33	8.068 (7.194–8.525)^a^		6.049 (4.527–7.052)^a^		7.301 (5.926–7.782)^a^	
>60	34	8.117 (7.367–8.588)^a^		6.090 (4.503–6.521)^a^		7.268 (6.048–7.502)^a^	
**Smoking index**			0.906		0.372		0.513
<400	48	8.072 (7.246–8.659)^a^		5.622 ± 1.435^b^		7.223 (5.891–7.525)^a^	
≥400	19	8.127 (7.490–8.504)^a^		5.985 ± 1.623^b^		7.316 (6.726–7.531)^a^	
**pTNM stage**			0.408		0.420		0.821
0~Ia	49	8.068 (7.105–8.552)^a^		6.037 (4.409–6.502)^a^		7.310 (5.630–7.518)^a^	
Ib/IIa	18	8.218 (7.367–8.662)^a^		6.276 (4.828–7.132)^a^		7.177 (6.102–7.808)^a^	
**Differentiation grade**			0.589		0.207		0.711
Poor	13	8.324 (8.029–8.506)^a^		6.193 (5.734–6.772)^a^		7.301 (7.078–7.535)^a^	
Moderate	39	8.017 (7.265–8.558)^a^		5.758 (4.462–6.465)^a^		7.230 (5.856–7.531)^a^	
Well	15	8.107 (6.763–8.598)^a^		6.360 (4.356–7.288)^a^		7.318 (5.365–8.037)^a^	

*NSCLC, non-small cell lung cancer*.

a *data are presented as median (Q25–Q75)*,

b *data are presented as mean ± SD*.

## Discussion

Lung cancer is the leading cause of cancer-related death in the world (1). The poor prognosis of lung cancer is mostly due to the advanced stage of the disease. Improving the early diagnostic rate of lung cancer is very important. With the help of CT, more patients with lung nodules can be found. However, CT has a relatively high false positive rate, which may diagnose benign nodules as lung cancer and lead to unnecessary anxiety and surgery (5, 6, 16). It is imperative to find novel biomarkers for the auxiliary diagnosis of lung cancer together with CT. In this study, three miRNAs were enrolled into a model to predict the nature of pulmonary nodules. Current literatures have shown that miRNAs may serve as novel potential biomarkers for cancers (17, 18). Novel potential biomarkers like miRNAs was non-invasive method which was more valuable. Invasive operations, such as bronchoscopy and VATS not only increase the treatment costs but also the time of treatment, and some patients need to be hospitalized. What is more, invasive operations can bring patients surgical trauma. Compared with invasive test, a blood test/imaging is repeatable, easy sample management, cost-effectiveness, and non-invasive.

Plasma miRNAs have been reported to be involved in the occurrence and progression of lung cancer (19). Many studies have demonstrated the high diagnostic efficiency of miRNAs for early cancer detection (20–22). Based on previous studies, the diagnostic value of single miRNA for diagnosing pulmonary nodules was still poor (23). Thus, we combined of three miRNAs (miR-146a, miR-200b, and miR-7) for analysis in our study. Different from the early studies focused on a single or a few miRNAs (18, 24), the result of our study revealed that combining miRNAs and other clinical imaging features can obtain better diagnosis efficacy than those approaches using a single miRNA. A single miRNA only revealed some aspects of tumorigenesis, the combination of miRNAs constituted a better indicator for tumor occurrence and progression. And the diagnostic efficiency of a panel of miRNAs is usually better than that of a single miRNA (25). In our study, three out of 10 candidate miRNAs (miR-17, -146a, -200b, -182, -221, -205, -7, -21, -145, and miR-210) were found to increase significantly in NSCLC patients compared with those of benign individuals. Which demonstrated miR-146a, miR-200b, miR-7 may be the potential biomarkers for early-stage NSCLC. Nevertheless, the expressions of the other candidate miRNAs may be different significantly between NSCLC and benign patients when the sample size increased. We did not find the relationships between the expression of miR-146a, miR-200b, miR-7, and clinical characteristics in early-stage NSCLC. However, future studies may be needed to conduct to test other stage NSCLC to determine the relationships between the expression of above miRNAs and clinical characteristics.

Regarding the diagnosis of the lung nodules, most clinicians prefer to evaluate the pretest probability using clinical experience and judgment. Under this situation, the pretest probability is easily affected by subjectivity, and the result is not reliable. In this study, we established a prediction model with a mathematical formula, to distinguish benign and malignant pulmonary nodules, which is more reliable, measurable. Because a combination of molecular biological information and imaging features reflects various aspects of tumorigenesis. And we can get a risk score through the mathematical formula which contributes to decrease subjective. For imaging findings, the Swensen model and the VA model were used to diagnose the nature of lung nodules by some clinicians (26, 27). In the Swensen model, 6 variables of clinical and imaging features were predictors of malignancy, including age, smoking history, history of cancer, nodule diameter, upper lobe location, and speculation (26). In our prediction model, 2 variables of clinical and imaging features were included. We found that nodules with speculation and pleural indentation are more likely to be malignant, which was consistent with previous studies (26). Unlike many early studies focused on using clinical and imaging features to distinguish benign and malignant pulmonary nodules (28, 29), we added the new potential biomarker- miRNAs into our prediction model and achieved a relatively good discriminant result. Which can also provide the theoretical foundation for further exploring the role of combining miRNAs and, clinical and imaging features in the diagnosis of lung nodules.

Reviewing the overall existing literature of biomarkers for distinguishing NSCLC from benign pulmonary nodules, we may apply plasma miRNAs, ctDNA, circulating cancer cell (CTC), together with existing tumor biomarkers (such as CEA, NSE, Cyfra21-1) to the early diagnosis of lung cancer in clinic in the future.

More and more patients with pulmonary nodules are discovered due to the popularity of LDCT. The management of pulmonary nodules remains challenging (30), and evaluating the pretest probability of malignancy of pulmonary nodules is important. The result of the present study discloses that this model can diagnosis early-stage NSCLC with relatively high sensitivity and specificity, which will help to improve the strategies for pulmonary nodule management. In face of a patients with pulmonary nodules, we can choose different treatment programs, such as surgery, needle biopsy, or watchful waiting, according to the risk score of the model in the future. Furthermore, the model can be used for early detection of lung cancer, which can help to improve the prognosis of lung cancer.

This study has several limitations. Above all, this is a retrospective study with a small sample size, which may lead to potential bias. Furthermore, the proportion of benign nodules patients was relatively low, and this may can reduce diagnostic efficiency. Finally, the predictive model appears to be validated using a separate small set of patients from the same institution. Conducting a large-scale, multicentric and prospective clinical study will be more valuable in the future.

In conclusion, we found that Plasma miRNA-146a, miRNA-200b, and miRNA-7 may be the potential biomarkers for the early diagnosis of lung cancer. Our prediction model can help to identify the nature of pulmonary nodules with a relatively high diagnostic efficiency. Which will serve for the detection for early-stage lung cancer and improve the management of pulmonary nodules.

## Data Availability Statement

All datasets generated for this study are included in the manuscript/supplementary files.

## Ethics Statement

The studies involving human participants were reviewed and approved by the Ethics Committee of Sun Yat-sen University Cancer Center (No. B2017-050). The patients/participants provided their written informed consent to participate in this study.

## Author Contributions

LZ and KX: conception and design. KX, WW, YW, GW, and ZH: provision of study materials or patients. KX, YC, XZ, YWu, and RZ: collection and assembly of data. KX, WW, and YW: data analysis and interpretation. All authors: manuscript writing and final approval of manuscript.

### Conflict of Interest

The authors declare that the research was conducted in the absence of any commercial or financial relationships that could be construed as a potential conflict of interest.
